# Providing Sexual Companionship for Resources: Development, Validation, and Personality Correlates of the Acceptance of Sugar Relationships in Young Women and Men Scale (ASR-YWMS)

**DOI:** 10.3389/fpsyg.2020.01135

**Published:** 2020-06-03

**Authors:** Béla Birkás, Norbert Meskó, András N. Zsidó, Dóra Ipolyi, András Láng

**Affiliations:** ^1^Medical School, University of Pécs, Pécs, Hungary; ^2^Institute of Psychology, University of Pécs, Pécs, Hungary

**Keywords:** acceptance of sugar relationships, scale development, validation, personality correlates, transactional sex

## Abstract

A sugar relationship is a transactional sexual relationship in which a younger partner (sugar baby/boy) offers companionship and sexual services to a much older partner (sugar daddy/mommy) in return for material compensation. One aim of the present study was to develop an attitude scale assessing young women’s and men’s acceptance of sugar relationships. Another aim was to explore the possible associations of the acceptance of sugar relationships with psychological functioning in an intimate partner relationship and in a sexual relationship and with certain socially undesirable personality traits. Two online studies were conducted with a total number of 2052 participants (1879 women; age = 18–28 years). The results show that the Acceptance of Sugar Relationships in Young Women and Men Scale (ASR-YWMS) is a reliable and valid measure of young people’s attitude toward sugar relationships. The studies revealed that young women’s and men’s accepting attitude toward sugar relationships was positively associated with unrestricted sociosexuality, a game-playing love style (Ludus), self-focused sexual motivation (Study 1; *N* = 319; 272 women and 47 men), and with socially undesirable traits such as Machiavellianism, subclinical psychopathy, and a borderline personality organization (Study 2; *N* = 1733; 1607 women and 126 men). These findings suggest that a relatively high level of acceptance of sugar relationships is part of a mating strategy focused on opportunities of maximizing resources. This utilitarian, risk-taking and exploitative attitude is characteristic to a fast life history strategy, and it is a fundamental organizing principle of psychological and sexual functioning in intimate partner relationships.

## Introduction

### Transactional Sex

Transactional sex describes transactions, in which one partner provides resources for the other in exchange for the latter’s immediate sexual availability. This presumably evolved, complex bio-psycho-social process affected mate choice throughout human history and even in modern populations. Differences in mate choice preferences related to sexual dimorphism (e.g., number of partners) are suggested to be an important factor in the emergence of this phenomenon (e.g., Buss and Schmitt, 1993, 2019; Conroy-Beam et al., 2019a, b; Walter et al., 2020). For instance, Trivers (1972) points out that women’s and men’s mate choice preferences show marked differences as a result of sex differences in reproductive biology (e.g., in the amount of energy invested in procreation). Since male (but not female) reproductive success was essentially influenced by the number of available sexual partners in the evolutionary past, maximizing the number of partners is still a characteristic sexual strategy among men. This short-term mating strategy (i.e., mating with the largest possible number of partners) is limited by the number of women available for casual sex in a given population. Furthermore, promiscuous sexual behavior potentially entail direct parental investment for women (due to pregnancy) but not for men. For this reason, men are more willing to engage in short-term sexual encounters (e.g., Schmitt, 2005; Buss and Schmitt, 2019).

Accordingly, one of the most common evolutionary explanations for men’s more pronounced motivation for engaging in transactional sex attributes this sex difference to men’s need for promiscuity (e.g., Symons, 1979; Burley and Symanski, 1981; Salmon, 2008). The sexual strategies theory (Buss and Schmitt, 1993, 2019) describes transactional sex (e.g., prostitution) as an extremely short-term mate choice strategy and draws a parallel between mate choice and marketing in terms of their underlying mechanisms (see Buss and Foley, 2019). Salmon (2008) goes as far as suggesting that since men are much more willing than women to engage in purely physical casual sexual encounters, and they actively seek such encounters, this preference is a clearly favorable condition for transactional sex.

Another line of research on the essential sex differences in mate choice preferences focuses on men’s marked attraction to relatively young women (e.g., Seto, 2017; Conroy-Beam and Buss, 2019). Heterosexual men are generally attracted to women in their twenties, independently of their own age, whereas very few men are exclusively attracted to very young or very old women (e.g., Kenrick and Keefe, 1992; Antfolk et al., 2015; Tripodi et al., 2015). This pattern has been demonstrated in modern Western and non-Western cultures (Buunk et al., 2001; Antfolk, 2017; Walter et al., 2020), and it is also supported by indirect evidence from hunter-gatherer societies (e.g., Early and Peters, 2000; Marlowe, 2004) and from pre-industrial societies (e.g., Dribe and Lundh, 2009). In sum, men are attracted to young women, while they have limited opportunity to engage in sexual activity with such women (depending on their own attractiveness, social status, the time and energy required by courtship, and the generally poor prospects for a satisfying long-term relationship with a much younger woman), thus they strive to fulfill their needs through transactional sex (e.g., Kenrick and Keefe, 1992; Ringdal, 1997; Buunk et al., 2001). Arunachalam and Shah (2008) reported earnings data of a sample of more than 4,000 Mexican and Ecuadorian sex workers. Although not discussed by the authors, their data show that female sex workers’ income precisely follows the age trend in fecundity: earnings are relatively low until they peak in the early to mid-twenties, which is followed by gradual decline. The association between income and fertility is corroborated by the finding that the same age-related trend in income is not shown by female non-prostitutes, whose income peaks in their late forties (Arunachalam and Shah, 2008). Similar association between age and other cues to female fertility have been found in a study of Polish prostitutes (Prokop et al., 2018): physical signs of fertility in female prostitutes predict the prices of sexual services, and women showing better signs of fertility offer their services at higher prices. A study conducted with American female escorts (a common euphemism for sexual services) found that younger women charged higher fees than their older counterparts (Griffith et al., 2016). The costs of sex are apparently higher for young escorts. These findings are consistent with those reported by Dunn (2018), who found that younger escorts advertising their sexual services online offered services at higher prices than older escorts. Similar findings have been reported by Sohn (2016a,b, 2017a,b), who studied Indonesian men’s preferred age of potential female partners, their sensitivity to signs of female fertility, and their preferences related to services provided by female prostitutes.

### Sugar Relationship

A sugar relationship is a form of transactional sexual relationship in which an older and wealthier partner (sugar daddy/mommy) provides material resources to a younger partner (sugar baby/boy) in return for her or his companionship (Nayar, 2016). Partners usually spend leisure time together, and sexual activity is only involved if both partners give their consent. Such transactional sexual relationships were quite common as early as centuries ago (Nelson, 1993). Nowadays, due to digital technology, potential partners are able to find each other more easily (e.g., Nayar, 2016; Botnen et al., 2018), and can maintain their privacy by using websites designated to arrange sugar relationship^1^^,^^2^^,^^3^. Sugar relationship are not banned by law in most Western countries even though the related legal issues have been fiercely debated not only from a legal viewpoint but also from social and moral perspectives (e.g., Miller, 2011; Motyl, 2012; Jones, 2014; Motz, 2014). One of the most debated legal issues is whether sugar relationships are to be considered a form of prostitution (e.g., Motz, 2014). In several countries, where prostitution (and active support for prostitution) is banned, the availability of web services designed for managing sugar relationships are not restricted or limited (e.g., Miller, 2011; Motyl, 2012). That is, these countries maintain a legal distinction between being involved in prostitution and being involved in a sugar relationship. Those protesting against sugar relationships on a moral basis target their efforts at eliminating this legal vacuum (e.g., Jones, 2014).

The nature of sugar relationship has been studied in various scientific approaches. A sociological study, for example, adopted a primarily descriptive approach to the motives underlying university students’ involvement in the sex industry (Sagar et al., 2016). Feminist research focuses on issues such as, for example, the role of power and agency in sugar relationship (e.g., Cordero, 2015). A study adopting an economic viewpoint found that a relatively large proportion of young women pursuing expensive university studies registered at websites designated to arrange sugar relationship, which are considered by the author as a form of human capital investment (Mixon, 2019).

Another line of research on “sex for compensation” phenomena is devoted to female university students’ involvement in the sex industry. Preble et al. (2019) consider sugar relationship between young university students and their older and wealthier partners as a transactional sexual relationship that provides better conditions for the former to pursue studies, a career, or a higher social status in general. Betzer et al. (2015) assessed a large number of university students and found that those engaging in transactional sex (i.e., sex work) received significantly less financial support from their families, used drugs more frequently (e.g., cocaine), and scored lower on the Agreeableness subscale of the Big Five Inventory than other participants. Other findings also revealed socially undesirable personality traits in university students involved in transactional sex (e.g., Edwards, 2017; Blum et al., 2018). Furthermore, studies conducted in economically developed East Asian countries highlight the importance of a materialistic worldview as a possible explanation for many young women’s willingness to provide sexual services to older men in exchange for material compensation (e.g., Cheung et al., 2016; Song and Morash, 2016; Krisch et al., 2019).

Accordingly, gaining better insight into the psychological aspects of sugar relationship (e.g., correlations with personality traits, love and sexual styles, etc.), is still wanting. One reason for this is that most participants in previous questionnaire and interview studies had not been directly involved in a sugar relationship or transactional sex. For example, in the study by Betzer et al. (2015), only 227 out of 4386 participants reported to have engaged in transactional sex, while 2998 had never been involved in a transactional relationship, and 1161 did not respond. Edwards (2017) assessed 820 participants, of which 94 reported to have at least once engaged in transactional sex. These findings suggest that female students involved in sugar relationship form a “hidden population,” which is not easily accessible for empirical research (Haeger and Deil-Amen, 2010).

### Love Styles, Sociosexuality and Sexual Motives

Previous studies exploring the psychological processes involved in man-woman relationships primarily focused on phenomena such as romantic love, sociosexuality and sexual motivation rather than on transactional sex. Therefore, the combination of these related theories is important to adequately contextualize the issues concerning the motivation for engaging in a sugar relationship.

Love styles can be described as systems of attitudes and beliefs characterized by different emotional tones, which are related to certain personality traits (Lee, 1988). Three primary love styles can be distinguished: Eros (passionate, romantic; seeking the ideal and perfect love), Ludus (playful, uncommitted love; looking at love as a game), and Storge (friendship evolves to love). Secondary love styles are balanced mixtures of the three primary styles: Pragma (practical, rational love) is a combination of Storge and Ludus, Mania (obsessive or addictive love) is a combination of Eros and Ludus, and Agape (altruistic or unselfish love), a combination of Eros and Storge. Thus, the model consists of a total of six different love types, which describe individual differences in love (e.g., Raffagnino and Puddu, 2018). This classification is relatively complex, comprehensive, and consistent with everyday experience and language (Hendrick et al., 2006). Frey and Hojjat (1998) found consistent positive correlations between a preference for commitment and all love attitudes apart from Ludus, which negatively correlated with commitment. These results were supported by other findings establishing a positive relationship between Ludus and unrestricted sociosexual orientation (e.g., Jonason and Kavanagh, 2010; Lee et al., 2013; Proyer et al., 2018; Smith et al., 2019).

The term sociosexuality originally referred to the diversity of male and female sexual behavior (Jonason et al., 2019). This initial construct was extended incorporating short-term and long-term mating interest, past sexual behavior (Jackson and Kirkpatrick, 2007) and the desire as a new component (Penke and Asendorpf, 2008). Accordingly, an instrument was developed to measure individual differences in attitudes toward casual sex and related forms of behavior (Simpson and Gangestad, 1991) and was subsequently supplemented with the desire component (Penke and Asendorpf, 2008). Previous studies on the psychological contexts of sociosexuality have revealed that men are more willing to engage in casual sex than women, which applies globally (Schmitt, 2005; Lippa, 2009). In addition, unrestricted socio-sexuality is positively associated with mating effort maximization, while restricted sociosexuality is more closely related to parental efforts (Valentova et al., 2020). Finally, unrestricted sociosexuality positively correlates with openness to casual sex among online dating site users (Hallam et al., 2018). However, a related study found that unrestricted sociosexuality was associated with participants’ willingness to engage in casual sex only when it was accompanied by a low level of commitment in a long-term relationship (Rodrigues and Lopes, 2017).

Sexual motives are commonly defined as the conscious and subjective reasons stated by men and women for engaging in sexual activity (e.g., Meston and Buss, 2007; Hatfield et al., 2012; Meston and Stanton, 2017). Sexual activity is closely related to several biopsychosocial factors and contextual elements of the relationship including the type and duration of the relationship, and the partners’ attachment styles (Meston and Stanton, 2017). A number of studies revealed sex differences in sexual motivation (e.g., Meston and Buss, 2007; Meston et al., 2009, 2019), suggesting, that women’s sexual activity is primarily based on relationship-related reasons, while men are more likely to have self-focused reasons. Armstrong and Reissing (2015) studied the associations between relationship type and sexual motivation, and they found more pronounced physical motives in those who preferred casual sexual relationships, while those currently having a committed relationship showed stronger emotional motives. Developing emotional attachment to a prospective partner is not a necessary condition for engaging in casual sex, since neither partner expects to be involved in a committed relationship offering long-term benefits. The Hungarian Short Form of the Reasons for Having Sex Questionnaire (YSEX?-HSF; Meskó et al., 2019) is a self-report instrument similar to the original questionnaire in item composition and slightly different in factor structure which characteristics support both, the cross-cultural universality of human sexual motivation and also the cultural diversity (reflected in the differences of the factor structure). Initial results with the YSEX?-HSF indicated, that self-centered and stress-related motives for sex were positively associated with unrestricted sociosexuality.

This suggests that one’s willingness to engage in a sugar relationship is part of a utilitarian orientation characterized by the (mutual) exploitation of partners, and by a sexual strategy based on the maximization of the number of partners.

### Aims of Study 1

To the authors’ knowledge, no instrument measuring the attitude toward engaging in a sugar relationship has been developed yet. Such an instrument would enable researchers to obtain a more detailed picture of the psychological aspects of sugar relationship by assessing samples of both directly involved individuals and the average population.

In consistence with the above discussed literature, the present research focused on the following two aims: (1) development of a self-report instrument measuring the acceptance of sugar relationships, and its validation with other measures of psychological functioning in intimate partner-, and sexual relationships; (2) verification of the hypothesis that the acceptance of sugar relationships is positively associated with self-beneficial behaviors in intimate partner relationships, a game-playing love style, and unrestricted sociosexuality.

## Study 1

### Hypotheses and Predictions

The aim of Study 1 was to test the validity of the ASR-YWMS. The validation process tested the following hypotheses.

#### Hypothesis 1

Based on the fundamental psychological characteristics of male and female mating preferences and strategies (e.g., Ohno, 1967; Trivers, 1972; Walsh, 1993), we hypothesized that men would have a more positive attitude toward sugar relationships than women.

#### Hypothesis 2

Based on the results of previous research exploring the relationship between transactional sex and materialistic values (e.g., Cheung et al., 2016; Song and Morash, 2016; Krisch et al., 2019), we hypothesized that individuals with a more positive attitude toward sugar relationships would have more materialistic, utilitarian and self-focused views on romantic relationships and sexuality compared to individuals with a less positive attitude toward sugar relationships. The following predictions were derived from the above hypothesis.

#### Prediction 1

Based on previous findings on multifactorial sexual motivation (e.g., Meston and Buss, 2007; Meskó et al., 2019), we hypothesized that individuals with a more positive attitude toward sugar relationships would view sex as a means to achieve personal or relational goals (as reflected in a positive association of the ASR-YWMS with personal goal attainment and sex as coping).

#### Prediction 2

Related to the former prediction, we expected individuals with a more positive attitude toward sugar relationships to view intimacy and commitment as unnecessary for getting involved in sexual relationships (as reflected in a negative association of the ASR-YWMS with relational motives).

#### Prediction 3

In line with previous findings on the psychological correlates of openness to casual sex (e.g., Schmitt, 2005; Penke and Asendorpf, 2008; Lippa, 2009), we expected individuals with a more positive attitude toward sugar relationships to have a more unrestricted sociosexual attitude (as reflected in a positive association of the ASR-YWMS with the SOI).

#### Prediction 4

Transactional sexual relationships generally lack the emotional intimacy characteristic of committed relationships (e.g., Scull, 2019) and so can be connected with previous findings about positive associations between a game-playing love style (Ludus) and unrestricted sociosexual orientation (e.g., Jonason and Kavanagh, 2010; Lee et al., 2013; Proyer et al., 2018; Smith et al., 2019). Accordingly, we hypothesized that individuals with a more positive attitude toward sugar relationships would view love as a source of pleasure without commitment (as reflected in a positive association between the ASR-YWMS and the Ludus love style).

#### Prediction 5

Based on previous findings on love styles, we hypothesized that individuals with a more positive attitude toward sugar relationships would view sexual attraction, commitment, and friendship as inessential ingredients of love (as reflected in a negative association of the ASR-YWMS with the Eros, Agape, and Storge love styles).

### Method

#### Item Generation and Selection

Item generation did not follow the procedure conventional in psychological research (e.g., Loevinger, 1957), but an existing questionnaire entitled the Acceptance of Cosmetic Surgery Scale (ACSS; Henderson-King and Henderson-King, 2005) was adapted for the purposes of the present study. This instrument measures the level of openness to cosmetic surgery, and it has been translated into several languages and used in hundreds of studies conducted in various cultures over the past 25 years (for an overview, see Meskó and Láng, 2019).

The choice of the instrument was based on the well-established empirical finding that the acceptance of cosmetic surgery is related to self-objectification (e.g., Calogero et al., 2010, 2014; Vaughan-Turnbull and Lewis, 2015; Choi and DeLong, 2019), which means treating one’s own body as an object with a detached attitude. Since several studies have found that one’s involvement in transactional sex is also related to self-objectification (Chen, 2016; Horley and Clarke, 2016; Gayathri et al., 2018; Endong, 2019; Maas et al., 2019), this latter was expected to function as a latent variable determining a positive association between one’s attitude toward cosmetic surgery and that toward transactional sex. The ACSS comprises three subscales, which structure was kept in the scale adapted for the acceptance of sugar relationships. One subscale of this latter assesses the *intrapsychic* component, that is, one’s perception of the potential personal benefits associated with engaging in a sugar relationship (e.g., “It makes more sense to be involved in a sugar relationship than to feel depressed for years by a bad financial situation”). Another subscale measures the *social* component, which reflects one’s perception of engaging in a sugar relationship as a normative behavior sanctioned by one’s immediate environment (e.g., friends) or the wider society (e.g., “If it was beneficial for my future life, I would consider engaging in a sugar relationship”). The third subscale measures the *consideration* component, which reflects one’s willingness to consider engaging in a sugar relationship despite potential unfavorable consequences also weighed before decision making (e.g., “If I knew that it did not entail disapproval or negative consequences, I would like to try a sugar relationship”).

That is, the items were generated by translating the 15 items of the ACSS (Henderson-King and Henderson-King, 2005) into Hungarian and then rewording each item in such a way that the original subject (i.e., cosmetic surgery) was replaced with sugar relationship (see the questionnaire in Appendix 1). The obtained instrument was entitled Acceptance of Sugar Relationships in Young Women and Men Scale (ASR-YWMS). Since it was hypothesized that younger and older parties would be driven by different motives to engage in a sugar relationship, another version of the instrument was also developed for an older age group (entitled Acceptance of Sugar Relationships in Older Men and Women Scale; ASR-OMWS). Although data were simultaneously collected in the two age groups, only the version developed for younger people and the related findings are presented in this paper. Findings on the older age group will be published in a separate paper.

Some of the items of the newly acquired questionnaire were highly similar. To address this issue, we decided to develop a shorter and, thus, a more feasible version of the ASR-YWMS. First, we used confirmatory factor analysis (CFA) to check the unidimensionality of the latent variables as the item response theory (IRT) requires unidimensional latent variables. The ACSS has three subscales, therefore we used the same model for the ASR-YWMS and analyzed the three subscales separately. For this purpose, we used the graded response model (GRM) of item response theory (Samejima, 1969) and examined the psychometric properties of each item. We selected items that had a very high (>1.7) discrimination ability (Baker, 2001). Then, we observed the item information functions (IIF) of these items and selected those with the highest values from each subscale. Some of the items had very similar IIF curves and were synonymous in meaning. From these, we selected the one that we thought had a better wording and represented the latent variable it was intended to measure better. During these analyses, data from the two studies presented in this paper were merged to obtain a larger sample (*N* = 2052, 1879 women and 173 men, age range: 18–28, *M* = 21.61, *SD* = 3.16; see further details in “Sample and Procedure” of this study and of Study 2) and, thus, a better accuracy.

**FIGURE 1:**
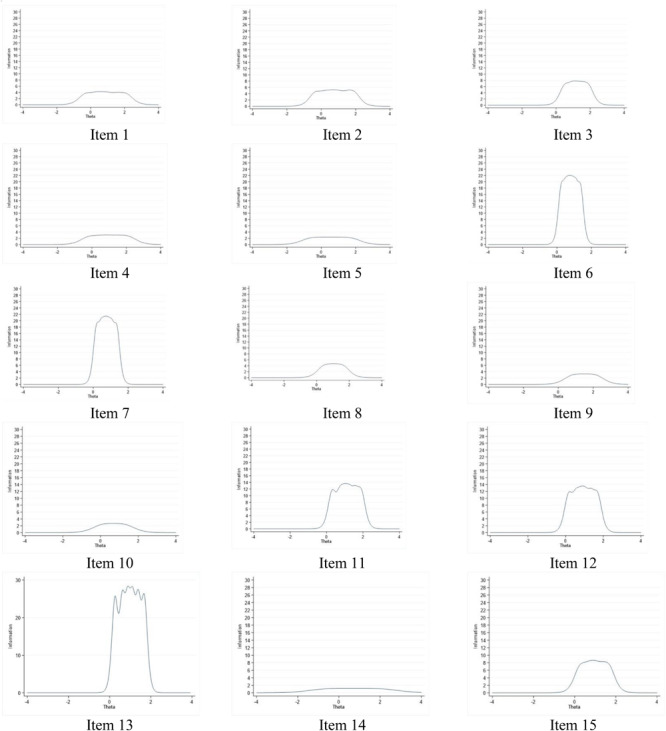
Item information curves for the 15 items of ASR-YWMS starting from **(top left)** item 1 to **(bottom right)** item 15.

The three-factor CFA (TLI = 0.969; CFI = 0.975; RMSEA = 0.070 [90% CI = 0.066 –0.074]; SRMR = 0.023) showed an excellent fit (Hu and Bentler, 1998). The GRM showed that all items have very high (>1.7) discrimination ability. Based on the IIFs, we selected items 1 and 2 from the first subscale (items 1, 2, 4, 5, and 14) but they were very similar, and thus, we decided to only use item 2 that also had a slightly better IIF (see Figure 1). We selected items 11, 12, and 13 from the second subscale (items 9, 11, 12, 13, and 15) but items 11 and 12 were very similar so we chose to drop item 11 and keep only items 12 and 13. Finally, we selected items 3, 6 and 7 from the third subscale (items 3, 6, 7, 8, and 10) but items 6 and 7, again, had close to identical IIFs, therefore we chose to retain items 3 and 7 only. Since only a small number of items remained after the analyses, keeping the original three-factor model would not be impractical. Thus, we propose to use the questionnaire with a single factor comprising all five items (nr 2, 3, 7, 12, and 13).

#### Sample and Procedure

After giving informed consent, 319 participants (272 women and 47 men) completed the questionnaires. Participants’ age ranged between 18 and 28 years (*M* = 24.64, *SD* = 2.77). Relationship status: currently single (19.4%); has casual relationships but no permanent partner (9.4%); is in a committed relationship/married but does not live with the partner (32.2%); is in a committed relationship/married and lives with the partner (39.2%). Registered at a dating site (11.0%); registered at a site designated to arrange sugar relationships (0.9%); registered at both types of sites (2.8%); not registered at either type of sites (85.3%). Currently involved in a sugar relationship (4.1%). Total number of sexual partners: 0 (3.1%), 1 (14.1%), 2 (11.3%), 3 (6.0%), 4 (6.3%), 5–6 (16.6%), 7–9 (13.5%), 10–19 (14.4%), 20 or more (14.7%). Place of residence: small village (4.7%); large village (2.5%); small/medium-sized town (11.6%); municipal town/city (23.8%); capital city and its agglomeration (57.4%).

Data were collected online. The survey was edited in Google Forms. The link to the survey was disseminated via Facebook and via one of the most popular and influential Hungarian internet portals, Index^4^. All participants gave informed consent, and none of them was rewarded for participation. The research plan received ethical approval from the Hungarian United Ethical Review Committee for Research in Psychology (Ref. No. 2018/115).

#### Instruments

##### Acceptance of sugar relationships in young women and men scale (ASR-YWMS)

The ASR-YWMS is the scale whose development, reliability analysis and validation were the objectives of the present study. The item generation procedure and sample items were presented previously, while the psychometric properties of the scale are discussed below. The scale contains five items (see Appendix 1). Cronbach’s α for the ASR-YWMS in the present study was 0.95.

##### Love attitudes scale, short form (LAS-SF; Hendrick et al., 1998)

The LAS-SF contains 24 items, which compose the following six subscales: Eros (erotic, romantic, passionate love style), Ludus (game-playing love style), Storge (affectionate, friendship-oriented love style), Pragma (rational, shopping-list love style), Mania (possessive, dependent love style), and Agape (selfless love style). Each subscale has four Likert items, and respondents indicate the extent to which each item applies to them on a 5-point rating scale ranging from 1 (strongly disagree) to 5 (strongly agree). Thus, higher scores reflect higher preference for the specific love style. Cronbach’s α values for the six subscales were as follows: Eros: 0.77; Ludus: 0.73; Storge: 0.88; Pragma: 0.60; Mania: 0.72; Agape: 0.83.

##### Sociosexual orientation inventory, revised (SOI-R; Penke and Asendorpf, 2008; adapted to hungarian by Meskó et al., 2014)

The SOI-R comprises nine items assessing one’s willingness to engage in uncommitted sexual encounters. The items compose three subscales measuring the three components of behavior, attitude and desire. Responses are given on 9-point rating scales (scale anchors vary across items). Higher scores on each subscale indicate more unrestricted sociosexuality in terms of behavior, attitude and/or desire. Cronbach’s α values for the three subscales and the overall scale were as follows: Behavior: 0.87; Attitude: 0.83; Desire: 0.90; SOI-R (overall): 0.88.

##### Reasons for having sex questionnaire, hungarian short form (YSEX?-HSF; Meskó et al., 2019)

The YSEX-HSF is a self-report instrument assessing sexual motivation. The Hungarian version differs from the original YSEX? four factor questionnaire. The scale comprises 73 items, which compose the following three subscales: Personal goal attainment, Relational reasons, Sex as coping. Each item is rated on a 5-point scale offering the following options: 1 = “None of my sexual experiences”; 2 = “Few (…)”; 3 = “Some (…)”; 4 = “Many (…)”; 5 = “All of my sexual experiences.” Thus, higher scores reflect higher levels of the measured sexual motive. Cronbach’s α values for the three subscales were as follows: Personal goal attainment: 0.91; Relational reasons: 0.92; Sex as coping: 0.91.

### Results

To test Hypothesis 1, we compared men’s and women’s scores on the ASRS with an independent samples *t*-test. Men (*M* = 17.11; *SD* = 9.80) – compared to women (*M* = 13.27; *SD* = 9.16) – scored significantly higher on the ASRS [*t*(317) = 2.622; *p* < 0.01] with a medium effect size (Hedges’ *g* = 0.41). Thus, results confirmed Hypothesis 1. Men tended to have a more positive attitude toward sugar relations than women.

The predictions of Hypothesis 2 were tested with Pearson’s correlation coefficients. Results of these analyses along with the means and standard deviations of the variables are shown in Table 1. Acceptance of sugar relationships showed a significant positive association with two sexual motives including personal goal attainment and sex as coping. Both correlations were moderate in magnitude. The importance of relational reasons for sexual relationships was unrelated to the acceptance of sugar relationships. Regarding sociosexuality, all subscales and thus the total score consistently showed significant positive associations with the acceptance of sugar relationships. All correlations were moderate in magnitude. Concerning love styles, the acceptance of sugar relationships was significantly associated with the Eros, Ludus, and Agape love styles. It showed a low negative correlation with Eros, a moderate positive correlation with Ludus, and a marginal negative correlation with Agape. Although the correlation between the acceptance of sugar relationships and Agape was statistically significant, its magnitude was considered negligible. The acceptance of sugar relationships was unrelated to any of the Storge, Pragma, and Mania love styles. In line with the results of Pearson’s correlations, a multiple linear regression analysis (Supplementary Material 1) confirmed sociosexuality and Ludus love style as having the strongest unique relationship with acceptance of sugar relationships.

**TABLE 1:** Association between acceptance of sugar relationships and criterion variables; results of Pearson’s correlations and means and standard deviations of the measured variables.

	**ASR- YWMS**	**YSEX?-HSF**			**SOI-R**				**LAS-SF**					
		**Personal Goal Attainment**	**Relational Reasons**	**Sex as Coping**	**Behavior**	**Attitude**	**Desire**	**Total**	**Eros**	**Ludus**	**Storge**	**Pragma**	**Mania**	**Agape**
ASR- YWMS	–	0.457**	–0.029	0.358**	0.438**	0.396**	0.459**	0.529**	−0.267**	0.543*	–0.016	–0.010	0.116	−0.154*
*M*	13.84	45.11	85.24	39.45	10.03	18.99	10.90	39.92	16.72	7.99	9.61	8.83	9.74	11.60
*SD*	9.34	15.61	21.38	14.38	6.33	7.10	6.44	16.14	2.97	3.79	4.91	3.42	3.86	3.97

*p < 0.01; **p < 0.001. ASR-YWMS, Acceptance of Sugar Relationships in Young Women and Men Scale. YSEX?-HSF, Why Have Sex – Hungarian Short Form. SOI-R, Sociosexual Orientation Inventory Revised Version. LAS-SF, Love Attitude Scale – Short Form.

In sum, the results confirmed Predictions 1, 3, and 4 of Hypothesis 2. Individuals with a more positive attitude toward sugar relationships tended to have sex out of self-focused reasons, and they also tended to use sex as a means of coping with distress or relational problems. Furthermore, they were less restricted in sociosexual orientation and more willing to engage in sexual relationships without commitment. This was also reflected in their views on love. Individuals with a more positive attitude toward sugar relationships tended to see love as a source of pure pleasure unrestricted by exclusivity. Prediction 5 of Hypothesis 2 was partly confirmed. Individuals with a more positive attitude toward sugar relationships viewed intimacy, security, and sexual aesthetics as inessential ingredients of love, whereas the subjective importance of friendship and commitment in a love relationship was unrelated to participants’ attitude toward sugar relationships. The results did not support Prediction 2 of Hypothesis 2. Individuals’ attitude toward sugar relationships was unrelated to the sexual motives of intimacy, love, and physical attraction.

### Discussion

Study 1 contributed some essential insights into the nature of sugar relationship. The findings suggest that individuals engaging in a sugar relationship approach to intimate partner relationships tend to have a more self-focused attitude toward sexuality and love, which is associated with the predomination of self-interest in their relationship-related motives. These results are consistent with previous findings that revealed a positive association between a game-playing love style (Ludus) and unrestricted sociosexual orientation (e.g., Jonason and Kavanagh, 2010; Lee et al., 2013; Proyer et al., 2018; Smith et al., 2019).

In the present study, young participants with a more accepting attitude toward sugar relationships appeared more willing to use sex as a means to attain personal goals or to cope with problems as compared to those with a less accepting attitude toward sugar relationships (H2, P1). However, no association was found between relational motives for having sex and the acceptance of sugar relationships (as opposed to the expected negative correlation; see H2, P2), which questions the assumption that young people are led by cold and calculating rationality (i.e., by a lack of need for intimacy) to engage in such transactional sexual relationships. It is possible that being together with an intimate partner as a reason for having sex is simply irrelevant in a sugar relationship. This possibility is supported by certain findings obtained in former studies (e.g., Ryan, 2019; Scull, 2019).

All three components of unrestricted sociosexuality (i.e., behavior/experience, attitude, and desire reflecting a preference for uncommitted sexual encounters) showed positive associations with the acceptance of sugar relationships (H2, P3). That is, young participants with a more accepting attitude toward transactional sex reported to have had more casual relationships, had more frequent fantasies of such encounters and appeared less disapproving of them as compared to those with a less accepting attitude toward sugar relationships. These findings are in line with recent studies that obtained similar results in relation to tertiary students’ involvement in sex work and in promiscuity (e.g., Okafor and Duru, 2010; Sanders and Hardy, 2015; Mensah, 2019).

Data on participants’ love-related psychological characteristics revealed a similar picture. Those showing a more accepting attitude toward sugar relationships reported a higher preference for the game-playing Ludus love style than those with a less accepting attitude toward transactional sex (H2, P4). By contrast, a negative correlation was obtained for the Eros love style: those more willing to accept sugar relationship were less likely to be involved in a passionate love relationship based on physical attraction as compared to those less approving of transactional sex (H2, P5). The acceptance of sugar relationships was not related to any of the other love styles including the friendship-oriented Storge, the practical and predictable Pragma, the obsessed and overwhelming Mania, and the selfless Agape. These love styles appear to be irrelevant in the context of transactional sexual relationships. Although no previous study focused specifically on the association between love and sugar relationship, comparable findings were obtained in studies examining the relationship between love and openness to uncommitted sex (e.g., Jonason and Kavanagh, 2010; Proyer et al., 2018; Jonason et al., 2019).

Finally, young men reported a more accepting attitude toward sugar relationships than young women (H1). Participants’ sexual orientation was not recorded in Study 1, therefore it was assumed that heterosexual participants formed the majority of the sample (in consistence with available data on the average population) (e.g., Gates and Newport, 2012; Lam, 2016). Accordingly, the obtained sex difference was interpreted in a heterosexual context, and as such it is in line with the predictions of the Sexual Strategies Theory (Buss and Schmitt, 1993). This theory suggests that men strive to maximize the number of sexual partners as a result of the increased reproductive success associated with this strategy in the evolutionary past. By contrast, maximizing the number of sexual partners did not directly increase women’s reproductive success. It is presumable, however, that young men involved in heterosexual sugar relationship do not approach older women with the implicit prospect of procreation. Therefore, the importance of this motive in sugar relationship is yet to be clarified.

Transactional sexual relationships appear to offer an adequate form of intimacy to individuals with a strong preference for short-term relationships enabling them to utilize exploitative interpersonal tactics. The findings of Study 1 on the relationship between sociosexuality and sexual motivation point to the possibility that an accepting attitude toward sugar relationships is part of a short-term mating strategy.

In summary, those participants of the present study showing relatively high acceptance of sugar relationships reported a preference for a game-playing, manipulative love style, unrestricted sociosexuality, and self-focused sexual motivation. These findings call for an empirical analysis of the relationship between the acceptance of sugar relationships and socially undesirable personality traits or personality organization, which was the subject of Study 2.

## Study 2

### Introduction

The findings of Study 1 raise the question of what personality traits may be associated with an accepting attitude toward sugar relationships. Openness to short-term relationships is known to be associated with specific patterns of personality traits. A related study found that unrestricted sociosexuality was positively associated with extraversion and negatively associated with agreeableness and conscientiousness (Schmitt and Shackelford, 2008). Another line of related studies explored prostituted women’s personality traits and psychopathological features and found that prostitutes scored higher on impulsive sensation seeking than the control group (O’Sullivan et al., 1996). Furthermore, Brody et al. (2005) found that adult female prostitutes were exposed to an increased risk of borderline personality disorder (BPD). In a similar vein, Tull et al. (2011) also reported that patients with BPD were more likely to be involved in prostitution than non-BPD patients.

Other studies revealed that women engaging in transactional sexual relationships showed clinical symptoms of antisocial/psychopathic personality disorder (Brody and Potterat, 2010; Edwards and Verona, 2016; Edwards, 2017). Moreover, Brody et al. (2005) explicitly suggest that due to the social conditions and psychodynamics characteristic to transactional sex, prostitutes meet virtually all diagnostic criteria of antisocial personality disorder as defined in the DSM-IV (American Psychiatric Association, 1994).

Several of these psychopathological features (e.g., low self-control, selfish interpersonal behavior) are associated with certain socially aversive personality traits collectively referred to as the Dark Triad (DT). The DT is a personality construct that includes three interrelated traits: Machiavellianism, subclinical psychopathy, and subclinical narcissism (Paulhus and Williams, 2002). There are several characteristics common to the three DT traits such as callousness, being manipulative (Jones and Paulhus, 2011), diminished self-control (Jonason and Tost, 2010), a more present-oriented time perspective (Birkás and Csathó, 2015), inability to delay gratification (Brumbach et al., 2009; Birkás et al., 2015), and being exploitative (McDonald et al., 2012). In a study conducted with 225 non-patient university students (130 females), Láng (2015) found a positive correlation between Machiavellianism and borderline personality organization.

Dark Triad traits were found to be positively correlated with various dimensions of short-term mating but not with long-term mating (Jonason et al., 2009). In a recent study, Collisson et al. (2019) examined a rather peculiar behavior: the researchers wanted to know how frequently women engaged in foodie calls (i.e., how often they set up a date with someone for a free meal), and what personality traits were associated with an accepting attitude toward such dates. The results revealed that 23–33% of the female participants had at least once engaged in a foodie call. Those having had related experience viewed foodie calls more socially acceptable than those without any experience. Both engaging in, and showing an accepting attitude toward, foodie calls positively correlated with the DT traits. More specifically, those women who were willing to offer companionship in return for a free dinner, while having no intention to have a second date with the male partner, showed relatively high levels of socially undesirable traits such as Machiavellianism and subclinical psychopathy. Furthermore, Brazil and Forth (2019) reported that female participants found men with higher levels of psychopathy more attractive than other men. The results showed that the observed men’s psychopathic tendencies were positively associated with sociosexuality, with specific factors of social intelligence, and with female participants’ desirability ratings when controlling for the men’s physical attractiveness. People with higher levels of DT traits are also more prone to lower their mate selection standards, by which they expand the choice of potential partners and improve their prospects for short-term relationships (Jonason et al., 2012).

The results of Study 1 showed that an accepting attitude toward sugar relationships was associated with a manipulative love style, self-focused sexual motivation, and a preference for short-term relationships. The acceptance of sugar relationships may also be associated with DT traits, particularly with Machiavellianism and psychopathy. Edwards (2017) revealed in an empirical analysis that female and male university students providing sexual services for material compensation showed higher levels of impulsive-antisocial traits (impulsivity, irresponsibility, sensation seeking) than those who did not engage in such activity. These findings are in line with those reported by Blum et al. (2018), who found that university students’ involvement in transactional sex was associated with high-risk sexual behavior and certain mental problems. Those willing to have sex for compensation showed higher levels of impulsivity, compulsive sexual behavior, anxiety and traumatization, and lower self-esteem. In sum, the findings suggest that those having sex for material compensation possess several socially undesirable personality traits and have various difficulties with social adaptation.

### Aims of Study 2

The aim of Study 2 was to test the expected associations between the acceptance of sugar relationships and socially aversive personality traits (e.g., Brody et al., 2005; Brody and Potterat, 2010; Tull et al., 2011; Edwards, 2017; Blum et al., 2018) and to replicate the previously obtained sex difference in the attitude toward sugar relationship (e.g., Buss and Schmitt, 1993, 2019; Gangestad and Simpson, 2000; Walter et al., 2020). In addition, we also expected to be able to confirm the unidimensional structure of the ASR-YWMS explored in Study 1.

Hypothesis 1 predicted that a similar sex difference would be observed in the acceptance of sugar relationships as that found in Study 1.

Hypothesis 2 included two predictions on the associations between the acceptance of sugar relationships and socially aversive personality traits. First, participants with a more positive attitude toward sugar relationship were expected to score higher on DT trait measures (i.e., subclinical narcissism, subclinical psychopathy, Machiavellianism; Prediction 1). Second, participants with a more positive attitude toward sugar relationships were expected to show severer symptoms of borderline personality organization (Prediction 2).

### Method

#### Sample and Procedure

After giving informed consent, 1733 participants (1607 women and 126 men) completed the questionnaires. Participants’ age ranged between 18 and 28 years (*M* = 21.05, *SD* = 2.91). Relationship status: currently single (37.8%); has casual relationships but no permanent partner (5.7%); is in a committed relationship/married but does not live with the partner (35.4%); is in a committed relationship/married and lives with the partner (21.2%). Registered at a dating site (11.1%); registered at a site designated to arrange sugar relationship (0.7%); registered at both types of sites (1.4%); not registered at either type of sites (86.8%). Total number of sexual partners: 0 (2.4%), 1 (13.5%), 2 (24.8%), 3 (15.6%), 4 (10.9%), 5–6 (9.4%), 7–9 (10.0%), 10–19 (7.7%), 20 or more (6.6%). Place of residence: small village (6.5%); large village (5.7%); small/medium-sized town (17%); municipal town/city (29.4%); capital city and its agglomeration (41.5%).

Data were collected online. The survey was edited in Google Forms. The link to the survey was disseminated via Facebook and via one of the most popular and influential Hungarian internet portals, Index^4^. All participants gave informed consent, and none of them was rewarded for participation. The research plan received ethical approval from the Hungarian United Ethical Review Committee for Research in Psychology (Ref. No. 2019/51).

#### Instruments

##### Acceptance of sugar relationships in young women and men scale (ASR-YWMS)

The ASR-YWMS is the scale whose development, reliability analysis and validation were the objectives of the present study. Cronbach’s α for the ASR-YWMS in the present study was 0.937.

##### Borderline personality inventory (BPI; Leichsenring, 1999)

The BPI is a 53-item self-report measure of borderline personality organization (BPO). Since the non-clinical sample of Study 2 was expected to show relatively mild features of BPO, the original “yes-no” response format of the BPI was replaced with four-point rating scales (ranging from “never” to “always”) more sensitive to subclinical psychopathology, thus the BPI was used as a Likert scale (for a previous application of this procedure, see Láng, 2015). The BPI measures four aspects of BPO: Identity Diffusion, Fear of Fusion, Primitive Defense Mechanisms, and Impaired Reality Testing. Since Study 2 only concerned BPO as a global construct, only the overall score and not the subscale scores on the BPI were included in the data analysis. The overall BPI scale showed high internal consistency (Cronbach’s α = 0.93).

##### Short dark triad (SD3; Jones and Paulhus, 2014)

The SD3 is a 27-item self-report instrument, whose three subscales measure three socially aversive personality traits: Machiavellianism (e.g., “Generally speaking, people won’t work hard unless they have to”), subclinical narcissism (e.g., “Many group activities tend to be dull without me”), and subclinical psychopathy (e.g., “It’s true that I can be nasty”). Each subscale comprises nine Likert items, and each item is rated on a 5-point scale. Cronbach’s α values for the three subscales were as follows: Machiavellianism: 0.75; Narcissism: 0.75; Psychopathy: 0.73.

### Results

The expected sex difference in the acceptance of sugar relationships was tested with an independent samples *t*-test. Men (*M* = 17.21, *SD* = 10.52) as compared to women (*M* = 11.44, *SD* = 7.85) scored significantly higher on the ASR-YWMS [*t*(136.123) = 6.029, *p* < 0.001] with a moderately large effect size (Hedges’ *g* = 0.71). That is, men reported a more positive attitude toward sugar relationships than women. This finding confirmed Hypothesis 1, and replicated the sex difference obtained in Study 1.

The predictions of Hypothesis 2 were tested with Pearson’s correlation coefficients (see Table 2). The results revealed significant positive associations between the acceptance of sugar relationships and all three socially aversive personality traits. The correlations obtained for Machiavellianism and subclinical psychopathy were low to moderate in magnitude, while narcissism showed a correlation of negligible strength. Thus, Prediction 1 of Hypothesis 2 was partly confirmed. Participants with a more positive attitude toward sugar relationships reported more pronounced Machiavellian and psychopathic traits, whereas narcissism appeared to be conceptually unrelated to the acceptance of sugar relationships. Prediction 2 of Hypothesis 2 was confirmed. Participants with a more positive attitude toward sugar relationships reported more pronounced features of borderline personality organization. In line with the results of Pearson’s correlations, a multiple linear regression analysis (Supplementary Material 2) conformed the unique relationship of all three dark personality traits and borderline personality organization with the acceptance of sugar relationships.

**TABLE 2:** Association between acceptance of sugar relationships and socially aversive personality traits; results of Pearson’s correlations and means and standard deviations of the measured variables.

	**ASR-YWMS**	**SD3**			**BPI**
		**Machiavellianism**	**Subclinical psychopathy**	**Subclinical narcissism**	
ASR-YWMS	–	0.351	0.349	0.191	0.267
*M*	11.86	27.34	19.82	23.75	78.22
*SD*	8.21	6.57	6.24	6.17	18.20

*All correlations are significant at the level of *p* < 0.001. ASR-YWMS, Acceptance of Sugar Relationships in Young Women and Men Scale. SD3, Short Dark Triad. BPI, Borderline Personality Inventory.*

### Discussion

The results of Study 2 confirmed both the expected factor structure of the ASR-YWMS (H1, P1) and the predicted sex difference in the acceptance of sugar relationships, that is, men scored significantly higher on the ASR-YWMS than women (H1, P2). Another important finding of Study 2 was the obtained positive association between the acceptance of sugar relationships and each of the assessed socially aversive personality traits (H2, P1). That is, participants reporting a more accepting attitude toward sugar relationship showed higher levels of Machiavellianism and subclinical psychopathy. These results are consistent with findings of related previous studies, which revealed that the DT traits were positively associated with a preference for short-term intimate partner relationships, and with women’s manipulative, amoral tactics related to female intrasexual competition (Honey, 2017; Semenyna et al., 2018, 2019).

The acceptance of sugar relationships was also positively associated with borderline personality organization (H2, P2). This suggests that a transactional sexual relationship is more likely to be endorsed by those who are particularly prone to show inadequate or extreme emotional reactions, to engage in impulsive behavior, and to be involved in intimate partner relationships that lack stability. This finding is in line with results of several previous studies revealing a relationship between borderline personality organization and sexual impulsivity (e.g., Sansone et al., 2008; Frías et al., 2016).

Importantly, the present study was conducted with a sine morbo sample, the majority of whom lived in a committed intimate partner relationship (in marriage or in a permanent relationship), and nearly 87% of whom were not registered at any sites designated to arrange dates or sugar relationship. In light of these sample characteristics, it is particularly interesting that positive associations were observed between the acceptance of sugar relationships and socially aversive traits such as Machiavellianism, subclinical psychopathy, and borderline personality organization. These findings suggest that those are the most likely to show interest in transactional sex who possess socially aversive personality traits to a certain extent.

In consistence with Study 1, the findings obtained in Study 2 also offer themselves for a more detailed explanation. Several studies revealed a close relationship between DT traits and mate choice preferences and strategies. These findings suggest that the forms of sexual behavior associated with DT traits generally fail to meet social norms and moral standards, and as such they are maladaptive. More specifically, socially aversive traits may also prove efficient in meeting certain adaptive challenges to mating and survival, while the potential advantages of these traits are primarily realized not through support provided for genetic relatives but through self-beneficial strategies such as the desire for status.

## General Discussion

In summary, the present studies revealed novel findings in several respects. On one hand, the Acceptance of Sugar Relationships in Young Women and Men Scale (ASR-YWMS) is the first questionnaire, to the authors’ knowledge, that has been designed to measure the acceptance of sugar relationships. On the other hand, the present paper is the first to empirically analysis of the attitude toward sugar relationship (transactional sex) in an evolutionary approach.

The ASR-YWMS provides a self-report measure of the acceptance of sugar relationships as a specific form of transactional sexual relationships among young adults who are willing to offer companionship and/or sexual services for material compensation. The questionnaire is a reliable and valid instrument showing the expected associations with intimate partner relationships, sexual motivation, and sociosexual orientation. The obtained findings revealed that the acceptance of sugar relationships was positively associated with a preference for short-term intimate partner relationships as reflected in unrestricted sociosexuality, self-focused (as opposed to relationship-focused) sexual motivation, and a game-playing, manipulative love style (as opposed to an eros-based love style, with which the acceptance of sugar relationships was negatively associated). Furthermore, the findings also revealed that an accepting attitude toward sugar relationship was positively associated with socially aversive personality traits such as manipulation (Machiavellianism), exploitation (subclinical psychopathy), and fear of intimacy combined with a general inner instability (borderline personality organization).

However, the two studies presented in this paper also touch upon issues outside the scope of the proximate factors underlying young women’s and men’s attitude toward sugar relationships. Namely, the above mentioned personality traits, relationship-related preferences, love styles and sexual motives appear to be interrelated elements of individual lifestyles or worldviews rather than a set of intrapsychic and interpersonal functions unrelated to each other. The findings suggest that an accepting attitude toward sugar relationships is part of a fundamentally goal-directed mating strategy focused on opportunities of maximizing resources. This utilitarian, short-term oriented, risk-taking and exploitative attitude appears to be a fundamental organizing principle of psychological and sexual functioning in intimate partner relationships.

### Limitations

It has to be noted that the obtained findings only provide indirect insights into the nature of sugar relationship, since only a small fraction of participants reported to have been directly involved in such relationships. For this reason, the ASR-YWMS should be used in further studies to assess the attitude of young people engaging in sugar relationship. In addition, the instrument should be completed with a behavioral measure to explore the association between transactional sexual behavior and the attitude toward sugar relationships.

Furthermore, the present studies only focused on one side of sugar relationship, that is, on those young people who offer companionship (sexual services) in return for resources. A more comprehensive understanding of the phenomenon requires a complementary analysis of the other side focusing on the psychological characteristics of those older people who provide resources for their young partners. This analysis will be published separately due to the limited scope of the present paper. Findings on both sides may contribute to a better understanding of the psychological factors and evolutionary mechanisms underlying the attitude toward sugar relationships.

## Conclusion

Keeping the above mentioned limitations in mind, the results of the development and validation of the ASR-YWM show that the self-report questionnaire provides a valid measure of young people’s willingness to engage in a sugar relationship. The obtained findings support the idea that openness to transactional sex (e.g., to sugar relationships) in young people is associated with a preference for short-term relationships, a playful love style, self-centered sexual motivation, and aversive personality traits such as subclinical psychopathy, Machiavellianism, and borderline personality organization. We have demonstrated that these associations may also be observed in the average population. One’s utilitarian mating strategy based on exploiting others and oneself probably is at play not only when one is currently involved in a sugar relationship. Future research is needed to understand what other psychological functions (e.g., self-esteem, attachment, motivation, etc.) may be associated with one’s willingness to engage in a sugar relationship. Furthermore, it is important to adequately clarify the significance of other evolved mating-related strategies (i.e., Life History Strategies) in one’s openness to sugar relationships. Most importantly, our study has shown that varying degrees of openness to sugar relationships in young individuals may be a fundamental part of mating psychology.

## Data Availability Statement

The datasets generated for this study are available on request to the corresponding author.

## Ethics Statement

The studies involving human participants were reviewed and approved by the Hungarian United Ethical Review Committee http://epkeb.ttk.hu/. The patients/participants provided their written informed consent to participate in this study.

## Author Contributions

NM, AL, and BB: conceptualization. NM and DI: methodology. AL and AZ: formal analysis and investigation. NM, BB, and AL: writing – original draft preparation. BB, AL, DI, and AZ: writing – review and editing. BB and NM: funding acquisition. NM and AL: resources. NM: supervision.

## Conflict of Interest

The authors declare that the research was conducted in the absence of any commercial or financial relationships that could be construed as a potential conflict of interest.

## Footnotes

1 https://www.seeking.com/

2 https://sugardaddie.com/

3 https://www.sugar-babies.com/

4 https://index.hu/
