# Supplementary material for: Providing Sexual Companionship for Resources: Development, Validation, and Personality Correlates of the Acceptance of Sugar Relationships in Young Women and Men Scale (ASR-YWMS)
Source: Front Psychol. 2020 Jun 3;11:1135. doi: 10.3389/fpsyg.2020.01135 (PMC7285874; doi:10.3389/fpsyg.2020.01135)
Supplement: Supplementary file 1 [file Data_Sheet_1.docx]

**Appendix 1**

Acceptance of Sugar Relationships in Young Women and Men Scale (ASR-YWMS)

A sugar relationship is a transactional sexual relationship in which an older and wealthier partner (sugar daddy/mommy) provides material resources to a younger partner (sugar baby/boy) in return for her or his companionship. Partners usually meet to spend leisure time together, and sexual activity is only involved if both partners give their consent.

Please indicate the extent to which you agree with each of the below statements using the seven-point rating scales ranging from (1) “absolutely disagree” to (7) “absolutely agree”.

| absolutely |  |  |  |  |  | absolutely |
| --- | --- | --- | --- | --- | --- | --- |
| disagree |  |  |  |  |  | agree |
| 1 | 2 | 3 | 4 | 5 | 6 | 7 |

| 1. It makes sense to engage in a sugar relationship rather than spending years feeling bad about your financial situation. | 1 2 3 4 5 6 7 |
| --- | --- |
| **2. A sugar relationship is a good thing because it can help the sugar baby/boy have a satisfactory financial situation.** | 1 2 3 4 5 6 7 |
| **3. In the future, I could end up engaging in a sugar relationship.** | 1 2 3 4 5 6 7 |
| 4. People who are very unhappy with their financial situation should consider engaging in a sugar relationship as one option. | 1 2 3 4 5 6 7 |
| 5. If sugar relationship can make someone happier with their financial situation, then they should try it. | 1 2 3 4 5 6 7 |
| 6. If I could engage in a sugar relationship without risking exposure, I would consider trying it. | 1 2 3 4 5 6 7 |
| **7. If I knew I would not incur negative judgment or consequences, I would like to try a sugar relationship.** | 1 2 3 4 5 6 7 |
| 8. I have sometimes thought about engaging in a sugar relationship. | 1 2 3 4 5 6 7 |
| 9. I would seriously consider engaging in a sugar relationship if my best friend thought it was a good idea. | 1 2 3 4 5 6 7 |
| 10. I would never engage in any kind of sugar relationship. | 1 2 3 4 5 6 7 |
| 11. I would think about engaging in a sugar relationship in order to have a better financial situation. | 1 2 3 4 5 6 7 |
| **12. If it would benefit my career, I would think about engaging in a sugar relationship.** | 1 2 3 4 5 6 7 |
| **13. I would seriously consider engaging in a sugar relationship if I thought it would help me have a better financial situation.** | 1 2 3 4 5 6 7 |
| 14. A sugar relationship can be a big benefit to people in terms of how they feel about their financial situation. | 1 2 3 4 5 6 7 |
| 15. If a short-term sugar relationship made me more satisfied with my financial situation, I would think about trying it. | 1 2 3 4 5 6 7 |

*Scoring guide:*

Reverse-scored item: 10

*Note:*

Bold items represent the 5-item version of ASR-YWMS
