# Supplementary material for: Providing Sexual Companionship for Resources: Development, Validation, and Personality Correlates of the Acceptance of Sugar Relationships in Young Women and Men Scale (ASR-YWMS)
Source: Front Psychol. 2020 Jun 3;11:1135. doi: 10.3389/fpsyg.2020.01135 (PMC7285874; doi:10.3389/fpsyg.2020.01135)
Supplement: Supplementary file 2 [file Data_Sheet_1.pdf]

Supplement 1. The unique relationship of the measured variables with acceptance of sugar relationships; results of a multiple linear regression analysis.

		β	VIF	R^2
YSEX?-HSF	Personal Goal Attainment	.055	2.777	
	Relational reasons	- .174*	1.408	
	Sex as coping	.166*	2.044	
SOI-R Total		.279**	2.289	
LAS-SF	Eros	.011	1.354	.41**
	Ludus	.283**	1.807	
	Storge	.011	1.031	
	Pragma	- .017	1.062	
	Mania	.057	1.233	
	Agape	- .085	1.220	

Note: VIF = vector inflation factor; * $p < .01$, ** $p < .001$. YSEX?-HSF = Why Have Sex - Hungarian Short Form. SOI-R = Revised Sociosexual Orientation Inventory. LAS-SF = Love Attitude Scale - Short Form.

Supplement 2. The unique relationship of the measured variables with acceptance of sugar relationships; results of a multiple linear regression analysis.

		β	VIF	R^2
	Machiavellianism	.194**	1.670	
SD3	Subclinical Psychopathy	.133**	2.054	.17**
	Subclinical Narcissism	.082*	1.269	
BPI		.142**	1.326	

Note: VIF = vector inflation factor; * p < .01, ** p < .001. SD3 = Short Dark Triad. BPI = Borderline Personality Inventory.
